# Adult height and risk of 50 diseases: a combined epidemiological and genetic analysis

**DOI:** 10.1186/s12916-018-1175-7

**Published:** 2018-10-25

**Authors:** Florence Y. Lai, Mintu Nath, Stephen E. Hamby, John R. Thompson, Christopher P. Nelson, Nilesh J. Samani

**Affiliations:** 1 Department of Cardiovascular Sciences, University of Leicester, Leicester, UK; 2 NIHR Leicester Biomedical Research Centre, Glenfield Hospital, Leicester, UK; 3 Department of Health Sciences, University of Leicester, Leicester, UK

**Keywords:** Adult height, Genetically determined height, Mendelian randomisation, Instrumental variables, Disease risk

## Abstract

**Background:**

Adult height is associated with risk of several diseases, but the breadth of such associations and whether these associations are primary or due to confounding are unclear. We examined the association of adult height with 50 diseases spanning multiple body systems using both epidemiological and genetic approaches, the latter to identify un-confounded associations and possible underlying mechanisms.

**Methods:**

We examined the associations for adult height (using logistic regression adjusted for potential confounders) and genetically determined height (using a two-sample Mendelian randomisation approach with height-associated genetic variants as instrumental variables) in 417,434 individuals of white ethnic background participating in the UK Biobank. We undertook pathway analysis of height-associated genes to identify biological processes that could link height and specific diseases.

**Results:**

Height was associated with 32 diseases and genetically determined height associated with 12 diseases. Of these, 11 diseases showed a concordant association in both analyses, with taller height associated with reduced risks of coronary artery disease (odds ratio per standard deviation (SD) increase in height OR_epi_ = 0.80, 95% CI 0.78–0.81; OR per SD increase in genetically determined height OR_gen_ = 0.86, 95% CI 0.82–0.90), hypertension (OR_epi_ = 0.83, 95% CI 0.82–0.84; OR_gen_ = 0.88, 95% CI 0.85–0.91), gastro-oesophageal reflux disease (OR_epi_ = 0.85, 95% CI 0.84–0.86; OR_gen_ = 0.94, 95% CI 0.92–0.97), diaphragmatic hernia (OR_epi_ = 0.81, 95% CI 0.79–0.82; OR_gen_ = 0.91, 95% CI 0.88–0.94), but increased risks of atrial fibrillation (OR_epi_ = 1.42, 95% CI 1.38–1.45; OR_gen_ = 1.33, 95% CI 1.26–1.40), venous thromboembolism (OR_epi_ = 1.18, 95% CI 1.16–1.21; OR_gen_ = 1.15, 95% CI 1.11–1.19), intervertebral disc disorder (OR_epi_ = 1.15, 95% CI 1.13–1.18; OR_gen_ = 1.14, 95% CI 1.09–1.20), hip fracture (OR_epi_ = 1.19, 95% CI 1.12–1.26; OR_gen_ = 1.27, 95% CI 1.17–1.39), vasculitis (OR_epi_ = 1.15, 95% CI 1.11–1.19; OR_gen_ = 1.20, 95% CI 1.14–1.28), cancer overall (OR_epi_ = 1.09, 95% CI 1.08–1.11; OR_gen_ = 1.06, 95% CI 1.04–1.08) and breast cancer (OR_epi_ = 1.08, 95% CI 1.06–1.10; OR_gen_ = 1.07, 95% CI 1.03–1.11). Pathway analysis showed multiple height-associated pathways associating with individual diseases.

**Conclusions:**

Adult height is associated with risk of a range of diseases. We confirmed previously reported height associations for coronary artery disease, atrial fibrillation, venous thromboembolism, intervertebral disc disorder, hip fracture and cancer and identified potential novel associations for gastro-oesophageal reflux disease, diaphragmatic hernia and vasculitis. Multiple biological mechanisms affecting height may affect the risks of these diseases.

**Electronic supplementary material:**

The online version of this article (10.1186/s12916-018-1175-7) contains supplementary material, which is available to authorized users.

## Background

Epidemiological studies have associated higher adult height with lower risk of mortality from coronary artery disease (CAD) and respiratory diseases [1–7] and increased risk of atrial fibrillation (AF) [8, 9], venous thromboembolism (VTE) [9–11], cancer and cancer in specific sites [1, 7, 12–16]. Although such studies have typically adjusted for age, sex and some socio-economic and behavioral risk factors, the observed associations may still be due to unmeasured confounding. Height itself is determined by genetics and early-life factors, such as nutrient availability, socio-economic circumstances and diseases [17–19], and some of these can themselves impact on risk of some diseases in later life. Precise reason(s) for the association of adult height with risk of these diseases are not known. In particular, it is unclear whether the associations are primary (due to shared biological pathways affecting both adult height and risk of diseases) and not due to confounding.

Genetic approaches (Mendelian randomisation) that use genetic variants as instrumental variables have been used to test for causal relationships between exposure and disease outcomes [20]. Genotypes are generated at conception with alleles randomly passed on from each parent and are independent of environmental and life style factors that can confound epidemiological analysis. Also, they cannot be altered by disease and therefore remove the possibility of reverse causality. Several recent studies have used a Mendelian randomisation approach to assess the relationship of height with selected diseases, including CAD [21, 22], stroke [22], VTE [23] and cancers [16, 24–28].

A recent genome-wide association studies (GWAS) meta-analysis involving 253,288 individuals of European ancestry by the GIANT consortium identified 697 height-associated variants which explain ~ 20% of the heritability of adult height [29]. Here, using this set of genetic variants and the breadth and scale of the UK Biobank [30], we comprehensively evaluated the associations of adult height with 50 diseases in multiple body systems in the same population using both traditional epidemiological and genetic approaches. We additionally undertook pathway analysis of the height-associated genes to explore potential biological processes underlying the associations.

## Methods

### Study design and setting

Details of the design of the UK Biobank have been reported elsewhere [30]. Briefly, the UK Biobank is a population-based longitudinal study that recruited ~ 500,000 participants aged 40–69 years during 2006–2010 from throughout the United Kingdom (UK). Participants were recruited by inviting just over 9 million individuals from central NHS registration databases in the appropriate age group and living within around 20–25 miles of 22 recruitment centres established in England, Scotland and Wales with an eventual participation rate of around 5%. Detailed data on sociodemographic, health status, family history and life style were collected via questionnaires. Standing height was measured by the Seca 202 device, and other physical measurements such as weight and blood pressure were measured at the assessment centres and biological samples were taken for further analysis.

The UK Biobank data have been linked to Hospital Episode Statistics (HES), as well as national death registries and cancer registries. HES data covers admissions to NHS hospitals in the UK between April 1997 and March 2015, with the Scottish data dating back as early as 1981. HES uses International Classification of Diseases ICD 9 and 10 to record diagnosis information, and OPCS-4 (Office of Population, Censuses and Surveys: Classification of Interventions and Procedures, version 4) to code operative procedures. Death registries include all deaths in the UK up to mid-2015, with both primary and contributory causes of death coded in ICD-10. Cancer registries cover registrations across the UK from 1970s to 2014 with diagnoses (coded in ICD9 and 10) acquired from a variety of sources including NHS and private hospitals, cancer screening programmes, cancer centres, hospices and nursing homes, general practices as well as HES, death certificates and Cancer Waiting Time (CWT) data.

The UK Biobank performed genome-wide genotyping using the Affymetrix UK BiLEVE Axiom array on first 50,000 participants as part of the BiLEVE study [31] and subsequently using the Affymetrix UK Biobank Axiom® array for the remaining cohort. The two arrays are very similar with over 95% overlap content. Details on genotyping, quality control and imputation methodology have been described elsewhere [32]. The assayed genotype data from both stages have been jointly imputed providing genome-wide genotypes on over 70 million SNPs for each subject.

### Study participants

We included 459,324 UK Biobank participants with genotype data who self-reported as having a white ethnic background. We excluded 985 participants with missing height and 22 participants whose height was > 4 standard deviation (SD) away from the mean (< 131.6 and > 205.6 cm). On the basis of the genetic data, we further excluded subjects because of uncertain gender (*n* = 354), genotype data quality (high missingness, QC failure, etc.) (*n* = 7025) and relatedness (kinship coefficient > 0.088) (*n* = 33,504). Altogether, we investigated 417,434 unrelated individuals of white ethnic background with valid height measures and genotype data.

### Disease definition

We examined 50 diseases covering six broad categories:

(i) Cardiovascular diseases—coronary artery disease (CAD), hypertension, peripheral vascular disease (PVD), heart failure (HF), atrial fibrillation (AF), venous thromboembolism (VTE), aortic valve stenosis (AS) and stroke
(ii) Musculoskeletal diseases—osteoarthritis, osteoporosis, gout, sciatica, and intervertebral disc disorder (IDD), and hip fracture
(iii) digestive diseases—liver cirrhosis, peptic ulcer, gastro-oesophageal reflux disease (GORD), irritable bowel syndrome (IBS), inflammatory bowel disease (IBD), gallstone, appendicitis, diaphragmatic hernia and inguinal hernia
(iv) Psychiatric and neurological diseases—anxiety disorder, depression, bipolar disorder, multiple sclerosis (MS), epilepsy, dementia and Parkinson’s disease
(v) Other non-neoplastic diseases—chronic obstructive pulmonary disease (COPD), asthma, diabetes, hyperthyroidism, hypothyroidism, glaucoma, cataract and vasculitis
(vi) Cancer including cancer overall and 11 specific sites—lung, colorectum, prostate, female breast, uterus, ovary, kidney, bladder, melanoma, non-Hodgkin lymphoma and leukemia

We defined cases using both self-reported and registry data and included both prevalent and incident cases. Case definition and the proportion of cases that were self-reported are shown in Additional file 1: Table S1. All diseases have a minimum of 1000 cases from 417,434 subjects in this study, providing 80% power to detect a relative 15% difference in disease risk (i.e. an odds ratio of at least 1.15 or 0.85) per one SD change in height at *α* = 0.001.

### Statistical analysis

#### Epidemiological analysis

**Fig. 1:**
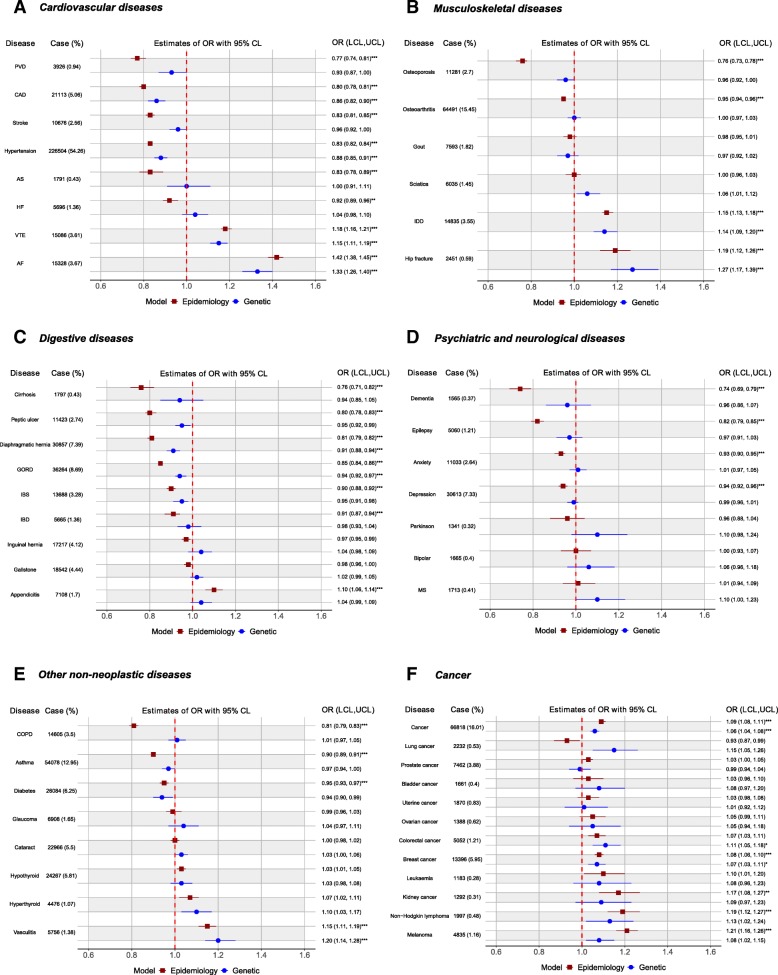
Epidemiological and genetic associations of height with diseases. Legend: Odds ratio (OR) and 95% confidence intervals per one standard deviation (SD) increase in height based on observed (epidemiology model) and genetically determined height (genetic model) are shown for **a** cardiovascular diseases (coronary artery disease (CAD), peripheral vascular disease (PVD), stroke, hypertension, aortic valve stenosis (AS), heart failure (HF), venous thromboembolism (VTE) and atrial fibrillation (AF)), **b** musculoskeletal diseases (osteoporosis, osteoarthritis, gout, sciatica, intervertebral disc disorder (IDD) and hip fracture), **c** digestive disorders (liver cirrhosis, peptic ulcer, diaphragmatic hernia, inguinal hernia, gastro-oesophageal reflux disease (GORD), irritable bowel syndrome (IBS), inflammatory bowel disease (IBD), gallstones and appendicitis), **d** psychiatric and neurological disorders (dementia, epilepsy, anxiety disorder, depression, bipolar disorder, Parkinson’s disease and multiple sclerosis (MS)), **e** other non-neoplastic diseases (chronic obstructive pulmonary disease (COPD), asthma, diabetes, glaucoma, cataract, hypothyroidism and hyperthyroidism and vasculitis), and **f** cancers and various sites. One SD is 9.2 cm; for men and women specific diseases, 1-SD corresponds to 6.8 cm and 6.2 cm, respectively. All epidemiological models were adjusted for age, sex, obesity (BMI ≥ 30), socio-economic status (Townsend deprivation index in highest quintile), Smoking status (ever smoker, exposed to environmental tobacco smoke, none), physical activity (vigorous exercise at least once a week or more) and other relevant disease-specific risk factors as described below: models for CAD—waist-hip-ratio, systolic blood pressure, use of insulin and family history of heart diseases; models for AF, VTE, PVD and heart failure—systolic blood pressure, use of insulin and family history of heart diseases; model for hypertension—use of insulin and family history of hypertension; model for stroke—waist-hip-ratio, systolic blood pressure, use of insulin and family history of stroke; model for COPD—family history of COPD; model for asthma—presence of hay fever or eczema; model for dementia—family history of dementia; depression—family history of depression; Parkinson’s disease—family history of Parkinson’s disease; model of glaucoma—systolic blood pressure and use of insulin; model for diabetes—waist-hip-ratio, systolic blood pressure and family history of diabetes; model of cataract—use of insulin; model for cancer overall—family history of lung/breast/prostate/bowel cancer; model for cancer of the breast—nulliparous, ever use of contraceptive pills, ever on hormone replacement therapy and family history of breast cancer; models for lung, prostate and colorectal cancers—family history of respective cancers. *p < 0.05, **p < 0.01, ***p < 0.001 after Bonferroni correction for 50 tests

We used logistic regression to estimate the association of height with risk of diseases, adjusted for age, sex, obesity (BMI ≥ 30 kg/m^2^), socio-economic status (based on quintiles of Townsend Deprivation Index [33] (an area-based measure of material deprivation calculated from census household data with a higher index indicating more deprived), smoking status (ever smoker, exposed to environmental tobacco smoke, none), physical activity and for individual diseases, other relevant factors, which included, where appropriate, waist-hip-ratio (WHR), systolic blood pressure (SBP), use of insulin, presence of hay fever/eczema and family history of relevant diseases, and for female diseases, parity, nulliparous, ever use of contraceptive pills and ever on hormone replacement therapy. Further details of the adjustments are given in the legend to Fig. 1.

#### Genetic analysis

We used a two-sample Mendelian randomisation (MR) approach to assess the association of genetically determined height with various diseases. Summary statistics for effect size of the height-associated SNPs were extracted from Wood et al*.* [29]. Of the 697 height-associated SNPs, 8 were unavailable in the genotype data imputed from the Haplotype Reference Consortium panel. Of the 691 variants serving as instrumental variables, 146 were genotyped and 545 were imputed with excellent imputation quality (mean 0.99 and the lowest 0.84). Effects of these height-associated SNPs on the risk of diseases were estimated with the UK Biobank data under an additive model adjusting for age, sex, array type (BiLEVE or main study) and five principal components. We calculated the ratio estimate for each height-associated variant [20] and then combined the estimates across all variants using meta-analysis [34]. For each height-associated variant, we extracted *β*_1_ (the effect size of the association between the variant and height) from published table [29], estimated *β*_2_ (the effect size of the association between the variant and the disease) under an additive mode of inheritance and computed *β*_3_ (the putative association between height and risk of disease mediated through that variant) using the equation

${\beta}_3=\frac{\beta_2}{\beta_1}$ and its standard error ${s}_3=\sqrt[]{\frac{1}{\beta_1^2{s}_2^{-2}}}$, where *s*_2_ is the standard error of *β*_2_.

Estimates of *β*_3_ across all the height-associated SNPs were then pooled using inverse-variance-weighted fixed-effect meta-analysis to obtain the overall association of genetically determined height on the risk of the disease [34]. In cases of high heterogeneity (*I*^2^ > 40%), we conducted a random-effects meta-analysis. The estimated *β*_3_ reflect the logarithm of the odds ratio of developing the disease per one SD increase in genetically determined height. We adjusted the *p* values for testing 50 diseases using Bonferroni correction in both epidemiological and genetic analyses.

#### Sensitivity analysis

The MR analysis assumes a linear relationship between height and risk of diseases. It relies on certain assumptions on the selected SNPs as instruments [20]: they are (1) associated with height, (2) not associated with confounding factors and (3) associated with the diseases only through their effect on height. We assessed the potential impact of violation of these assumptions using MR-Egger regression [35] and median-based methods [36] as sensitivity analysis. Standard MR analysis is equivalent to performing a weighted regression of the effect sizes of variant-disease associations (*β*_2_) against the effect sizes of variant-height associations (*β*_1_) with no intercept term, but MR-Egger conducts such regression with an unconstrained intercept. The estimated intercept in the MR-Egger regression [35] can be interpreted as an estimate of the average pleiotropic effects across the genetic variants. A non-zero intercept is indicative of directional pleiotropy, that is, the pleiotropic effects do not cancel out resulting in a biased MR estimate. We also conducted three median-based methods [36]: (1) simple median method which takes the median *β*_3_ estimate assuming all variants carry equal weight, (2) weighted median method which gives more weight to variants with more precise estimates and (3) penalised weighted median method which down-weights the contribution of heterogeneous variants. MR-Egger regression can give consistent estimates even when 100% of variants are invalid but requires the variants to satisfy a weaker assumption (the InSIDE assumption), whilst the weighted median methods can give consistent estimates as long as at least 50% of the weight comes from variants without pleiotropic effects. Additionally, we examined the association of genetically determined height with risk factors and repeated the genetic analysis excluding SNPs that showed a nominal association (*p* < 0.05) with suggested confounding factors. We restricted these sensitivity analyses to diseases showing evidence of association in primary genetic analysis (Bonferroni adjusted *p* < 0.05).

In this study, we defined cases using both self-reported and registry (hospital episodes, cancer and death registries) information. To assess any impact of including self-reported data, we repeated the epidemiological and genetic analyses defining cases based on registry data only. Furthermore, we defined cases including both prevalent and incident cases and have assumed that there were little changes in exposure of risk factors over time in the epidemiological analysis. To assess any impact of this assumption, we repeated the epidemiological analysis defining cases based on incidence only.

#### Genetic score for height

We calculated a genetic score for height to evaluate the effect of inheriting number of height-increasing alleles on risk of diseases. For each variant, we computed a score based on the posterior probabilities for the height-increasing allele, which is then multiplied by the effect size for height estimated by Wood et al. [29]. The genetic score is the sum of these weighted values across all 691 height-associated variants. We used regression analysis to assess the proportion of variance of height that can be explained by the genetic score. We divided the study subjects into quartiles based on their genetic scores with quartile 1 (Q1) carrying the least number of height-increasing alleles and Q4 carrying the most number of height-increasing alleles. We then used the Cochran-Armitage trend test to assess the presence of diseases across quartiles, and logistic regression to estimate the ORs for the quartiles.

#### Pathway analysis

To identify possible shared biologic processes between height and diseases, we identified pathways represented by the 691 height-associated SNPs using the Ingenuity Pathway Analysis Software (analysis performed on September 19, 2017). This analysis requires the assignment of each height-associated SNP to a specific gene. We selected the genes identified by Wood et al. [29] through their extensive bioinformatics analysis of each locus. From the pathways identified, we selected those that contained at least five genes from among the height-related genes and tested the association of height and risk of diseases through the specific pathway by combining disease-specific *β*_3_ estimates for all height variants in the pathway using meta-analysis. The results were not adjusted for multiple testing as this analysis was exploratory.

## Results

Of the 417,434 people included in the study, 54.0% were women, the mean age at recruitment was 56.8 years (range 38–73), and the mean height was 168.7 cm (SD 9.2). Taller people were younger, more likely to have a lower BMI, a lower WHR, and lower blood pressure (Table 1). They were also less likely to have ever smoked or be socio-economically deprived and more likely to be physically active. Taller women were more likely to be nulliparous and to have ever used the oral contraceptive pill and less likely to have ever been on hormone replacement therapy. Given that taller women were younger, this observation is likely to be confounded by age.

**Table 1:** Baseline characteristics of participants in the UK Biobank by quartiles of adult height

Characteristic	Height (cm)				Overall
	Quartile 1	Quartile 2	Quartile 3	Quartile 4	
Height (cm)—female	132–< 158	158–< 163	163–< 167	167–199	132–199
Height (cm)—male	132–< 171	171–< 176	176–< 180	180–205	132–205
N	87,358	117,771	97,416	114,889	417,434
Age	58.8 (7.5)	57.5 (7.8)	56.4 (7.9)	54.9 (8.1)	56.8 (8.0)
Sex—female	52.8%	55.7%	55.7%	51.6%	54.0%
Body mass index (BMI)	28.1 (4.9)	27.6 (4.8)	27.2 (4.7)	26.8 (4.6)	27.4 (4.8)
Obese (BMI ≥ 30)	28.9%	25.4%	23.0%	20.4%	24.2%
Waist-hip-ratio (WHR)	0.88 (0.1)	0.87 (0.1)	0.87 (0.1)	0.87 (0.1)	0.87 (0.1)
Townsend deprivation index^a^	22.5%	18.1%	16.7%	16.1%	18.1%
Ever smoker	47.1%	46.2%	45.5%	45.5%	46.0%
Vigorous activity^b^	55.5%	58.8%	60.6%	63.0%	59.7%
Systolic BP (mmHg)	145.3 (21.3)	142.3 (20.8)	140.1 (20.5)	137.8 (19.5)	141.2 (20.7)
Diastolic BP (mmHg)	85.4 (11.3)	84.6 (11.3)	84.0 (11.3)	83.4 (11.2)	84.3 (11.3)
Female only					
Nulliparous	15.9%	17.3%	19.0%	22.7%	18.8%
Ever oral contraceptive	78.1%	81.3%	83.3%	84.9%	82.0%
Ever on hormone replacement therapy	45.0%	41.6%	38.1%	33.4%	39.3%

Data expressed as mean (SD) for continuous variables or as percentages for categorical variables; missing data—BMI (*n* = 464), WHR (*n* = 162), systolic BP (*n* = 348), diastolic BP (*n* = 346), Townsend deprivation index (*n* = 489), smoking status (*n* = 1481), physical activity (*n* = 547), nulliparous (*n* = 143), oral contraceptive (*n* = 34) and hormone replacement therapy (*n* = 87)

*BP* blood pressure

^a^Townsend deprivation index—highest quantile

^b^Vigorous activity—at least once a week for 10+ min

As expected, people carrying more height-increasing alleles are taller (Table 2). Regression analyses showed that the weighted genetic score explained 16.7 and 16.5% of variation of height for women and men, respectively. Individuals in the upper quartiles were marginally older, with similar sex composition across quartiles, and appeared to be associated with a slightly lower BMI, lower blood pressure and lower Townsend Deprivation Index, but not with smoking history, or undertaking vigorous physical activity. Women with higher genetic score are more likely to be nulliparous and ever on hormone replacement therapy.

**Table 2:** Characteristics of participants in the UK Biobank by quartiles of weighted genetic score for height

Characteristic	Weighted genetic score for height^a^			
	Quartile 1	Quartile 2	Quartile 3	Quartile 4
N	90,170	107,870	109,390	110,004
Height (cm)—female	159.2 (5.7)	161.6 (5.6)	163.3 (5.7)	165.8 (6.0)
Height (cm)—male	172.1 (6.2)	174.7 (6.2)	176.6 (6.2)	179.3 (6.5)
Age	56.7 (8.0)	56.8 (8.0)	56.8 (8.0)	56.9 (7.9)
Sex—female	53.9%	54.0%	53.8%	54.2%
Body mass index (BMI)	27.6 (4.9)	27.5 (4.8)	27.3 (4.7)	27.2 (4.7)
Obese (BMI ≥ 30)	25.5%	24.5%	23.8%	23.1%
Waist-hip-ratio (WHR)	0.87 (0.09)	0.87 (0.09)	0.87 (0.09)	0.87 (0.09)
Townsend deprivation index^b^	19.5%	18.2%	17.8%	17.4%
Ever smoker	46.2%	46.2%	45.8%	45.9%
Vigorous activity^c^	59.4%	59.7%	60.1%	59.4%
Systolic BP (mmHg)	141.7 (20.9)	141.4 (20.7)	141.0 (20.6)	140.6 (20.5)
Diastolic BP (mmHg)	84.6 (11.3)	84.4 (11.3)	84.2 (11.2)	84.1 (11.3)
Female only				
Nulliparous	18.7%	18.4%	18.7%	19.5%
Ever contraceptive pill	81.9%	82.0%	82.2%	82.0%
Ever on hormone replacement therapy	38.8%	39.2%	39.4%	39.8%

Data expressed as mean (SD) for continuous variables or as percentages for categorical variables; missing data—BMI (*n* = 464), WHR (*n* = 162), systolic BP (*n* = 348), diastolic BP (*n* = 346), Townsend deprivation index (*n* = 489), smoking status (*n* = 1481), physical activity (*n* = 547), nulliparous (*n* = 143), oral contraceptive (*n* = 34) and hormone replacement therapy (*n* = 87)

*BP* blood pressure

^a^Quartile 1 of the genetic score carrying the least number and quartile 4 the most number of height-increasing alleles

^b^Townsend deprivation index—highest quantile

^c^Vigorous activity—at least once a week for 10+ min

### Association of height with diseases based on epidemiological and genetic analyses

The estimated odds ratio (OR) per one SD (9.2 cm) increase in height (epidemiological analysis) and genetically determined height (genetic analysis) are shown in Fig. 1. For men and women specific diseases, 1-SD of height corresponds to 6.8 and 6.2 cm respectively. Overall, 39 and 23 diseases showed evidence suggestive of epidemiological and genetic associations with height (*p* < 0.05), respectively. The height association remained for 32 and 12 diseases (11 in common) after adjusting for multiple testing.

Among cardiovascular diseases, we found inverse epidemiological associations of height with CAD, PVD, stroke, hypertension, AS and HF with an estimated OR between 0.77 (PVD) and 0.92 (HF) (Fig. 1a). With genetic analyses, we found strong evidence for inverse association for CAD (OR = 0.86, 95% CI 0.82–0.90, *p* < 0.0001) and hypertension (OR = 0.88, 95% CI 0.85–0.91, *p* < 0.0001). In contrast to these inverse associations, taller height was associated with increased risk of VTE and AF in both epidemiological (VTE: OR = 1.18, 95% CI 1.16–1.21, *p* < 0.0001; AF: OR = 1.42, 95% CI 1.38–1.45, *p* < 0.0001) and genetic analyses (VTE: OR = 1.15, 95% CI 1.11–1.19, *p* < 0.0001; AF: OR = 1.33, 95% CI 1.26–1.40, *p* < 0.0001).

Among musculoskeletal diseases, taller stature was strongly associated with an increased risk of IDD (epidemiological OR = 1.15, 95% CI 1.13–1.18, *p* < 0.0001; genetic OR = 1.14, 95% CI 1.09–1.20, *p* < 0.0001) and hip fracture (epidemiological OR = 1.19, 95% CI 1.12–1.26, *p* < 0.0001; genetic OR = 1.27, 95% CI 1.17–1.39, *p* < 0.0001) (Fig. 1b). The observed inverse associations of height with osteoporosis (OR = 0.76, 95% CI 0.73–0.78, *p* < 0.0001) and osteoarthritis (OR = 0.95, 95% CI 0.94–0.96, *p* < 0.0001) in epidemiological analyses were attenuated in genetic analyses (osteoporosis: OR = 0.96, 95% CI 0.92–1.00; osteoarthritis: OR = 1.00, 95% CI 0.97–1.03).

Among digestive diseases (Fig. 1c), we found inverse epidemiological associations between height and risks of liver cirrhosis, peptic ulcer, diaphragmatic hernia, GORD, IBS and IBD, with an estimated OR ranging from 0.76 to 0.91. For genetic analyses, the inverse height association was observed for diaphragmatic hernia (OR = 0.91, 95% CI 0.88–0.94, *p* < 0.0001) and GORD (OR = 0.94, 95% CI 0.92–0.97, *p* = 0.0001) only. We also observed epidemiological association of height with appendicitis (OR = 1.10, 95% CI 1.06–1.14, *p* < 0.0001), but the association was attenuated in genetic analysis (OR = 1.04, 95% CI 0.99–1.09).

Among psychiatric/ neurological diseases, we observed inverse epidemiological association for dementia, epilepsy, anxiety and depression; however, the corresponding genetic associations were much weakened (Fig. 1d). Similarly, the inverse associations observed for COPD, asthma and diabetes in epidemiological analysis were not found in genetic analysis (Fig. 1e). However, we observed taller height being associated with increased risks of vasculitis in both epidemiological (OR = 1.15, 95% CI 1.11–1.19, *p* < 0.0001) and genetic analyses (OR = 1.20, 95% CI 1.14–1.28, *p* < 0.0001).

Both epidemiological (OR = 1.09, 95% CI 1.08–1.11, *p* < 0.0001) and genetic analysis (OR = 1.06, 95% CI 1.04–1.08, *p* < 0.0001) showed evidence of a positive association of height with overall cancer (Fig. 1f). Of the 11 sites, height was associated with 8 and 5 sites at *p* < 0.05 (unadjusted) in epidemiological and genetic analyses, respectively. After adjusting for multiple testing, epidemiological association remained for four sites: female breast (OR = 1.08, 95% CI 1.06–1.10, *p* < 0.0001), kidney (OR = 1.17, 95% CI 1.08–1.27, *p* = 0.0053), non-Hodgkin lymphoma (OR = 1.19, 95% CI 1.12–1.27, *p* < 0.0001) and melanoma (OR = 1.21, 95% CI 1.16–1.26, *p* < 0.0001), and genetic association remained for two sites: breast (OR = 1.07, 95% CI 1.03–1.11, *p* = 0.0366) and colorectum (OR = 1.11, 95% CI 1.05–1.18, *p* = 0.0307).

### Sensitivity analysis

For the 12 diseases showing an association with genetically determined height, the intercept tests from the MR-Egger regression revealed little evidence for pleiotropy (Table 3). The associations revealed by MR-Egger regression and median-based methods were broadly similar to the primary genetic analyses for the nine non-neoplastic diseases. For cancer overall, the OR estimates from MR-Egger and weighted median methods remained similar to the primary genetic analysis. For breast cancer and colorectal cancer, the sensitivity analysis appears to suggest a weaker or null genetic association.

**Table 3:** Association between genetically determined height and risks of diseases based on inverse-variance-based, MR-Egger and median-based approaches

Disease	Inverse-variance-based method	*p* value intercept (MR-Egger)	MR-Egger	*p* value intercept (robust MR-Egger)	Robust MR-Egger	Simple median	Weighted median	Penalised weighted median
CAD	0.86 (0.82–0.90)	0.163	0.93 (0.84–1.03)	0.500	0.89 (0.80–1.00)	0.87 (0.83–0.92)	0.88 (0.83–0.93)	0.88 (0.83–0.93)
Hypertension	0.88 (0.85–0.91)	0.224	0.92 (0.86–0.99)	0.138	0.96 (0.88–1.04)	0.90 (0.88–0.93)	0.91 (0.88–0.93)	0.91 (0.88–0.93)
AF	1.33 (1.26–1.40)	0.444	1.39 (1.24–1.56)	0.436	1.40 (1.23–1.60)	1.36 (1.28–1.44)	1.34 (1.26–1.42)	1.34 (1.26–1.43)
VTE	1.15 (1.11–1.19)	0.147	1.23 (1.11–1.36)	0.434	1.18 (1.06–1.32)	1.14 (1.08–1.21)	1.18 (1.11–1.24)	1.16 (1.10–1.23)
GORD	0.94 (0.92–0.97)	0.595	0.93 (0.87–1.00)	0.695	0.94 (0.86–1.02)	0.96 (0.92–1.00)	0.95 (0.91–0.99)	0.96 (0.92–0.99)
Diaphragmatic hernia	0.91 (0.88–0.94)	0.516	0.88 (0.81–0.96)	0.813	0.91 (0.83–1.00)	0.94 (0.90–0.98)	0.93 (0.89–0.97)	0.93 (0.89–0.97)
IDD	1.14 (1.09–1.20)	0.041	1.29 (1.15–1.45)	0.198	1.22 (1.08–1.38)	1.14 (1.07–1.20)	1.13 (1.06–1.20)	1.12 (1.05–1.19)
Hip fracture	1.27 (1.17–1.39)	0.104	1.52 (1.20–1.92)	0.134	1.48 (1.17–1.87)	1.24 (1.08–1.42)	1.23 (1.07–1.42)	1.23 (1.07–1.41)
Vasculitis	1.20 (1.14–1.28)	0.139	1.33 (1.15–1.54)	0.240	1.32 (1.11–1.57)	1.21 (1.10–1.32)	1.22 (1.12–1.34)	1.22 (1.11–1.33)
Cancer overall	1.06 (1.04–1.08)	0.514	1.04 (0.99–1.10)	0.536	1.04 (0.99–1.09)	1.05 (1.02–1.09)	1.05 (1.02–1.08)	1.05 (1.02–1.08)
Colorectal cancer	1.11 (1.05–1.18)	0.234	1.02 (0.86–1.20)	0.173	0.99 (0.85–1.16)	1.10 (1.00–1.21)	1.06 (0.96–1.16)	1.04 (0.95–1.15)
Breast cancer	1.07 (1.03–1.11)	0.573	1.10 (0.98–1.23)	0.930	1.06 (0.94–1.19)	1.03 (0.97–1.10)	1.04 (0.98–1.11)	1.02 (0.96–1.09)

Association is expressed as odds ratios per 1 standard deviation increase in genetically determined height and its 95% confidence interval

*CAD* coronary artery disease, *AF* atrial fibrillation, *VTE* venous thromboembolism, *GORD* gastro-oesophageal reflux disease, *IDD* intervertebral disc disorder

The intercept term in MR-Egger regression can be interpreted as an estimate of the average pleiotropic effect across the genetic variants, with a non-zero intercept indicative of directional pleiotropy. MR-Egger uses standard regression in the analysis, whilst robust MR-Egger uses robust regression that down-weights the influence of outliers. The median-based method calculates a median of the causal estimates across all SNPs. The simple method calculates the simple unweighted median, the weighted method calculates the median using the inverse-variance weights, and the penalised method calculates the median down-weighting heterogeneous variants

We found that genetically determined height was associated with obesity, blood pressure, Townsend Deprivation Index and nulliparity (Additional file 2: Table S2). We repeated the genetic analyses excluding SNPs that were associated with these factors. This showed little impact on the estimates of the genetic associations, apart from the anticipated effect when excluding SBP-associated SNPs, which weakened the genetic association for hypertension. (Additional file 2: Table S3).

The estimates for using registry-based cases only were similar to that for using both self-reported and registry-based cases (Additional file 2: Table S4). This suggests little impact on using self-reported data. In addition, we repeated the epidemiological analysis excluding prevalent cases at baseline. There were no significant changes in the estimates, except for IDD in which the association appeared to have become much weakened (Additional file 2: Table S5). The confidence intervals for the estimates as expected became wider due to reduced statistical power.

### Genetic score of height and odds ratios of diseases

**Fig. 2:**
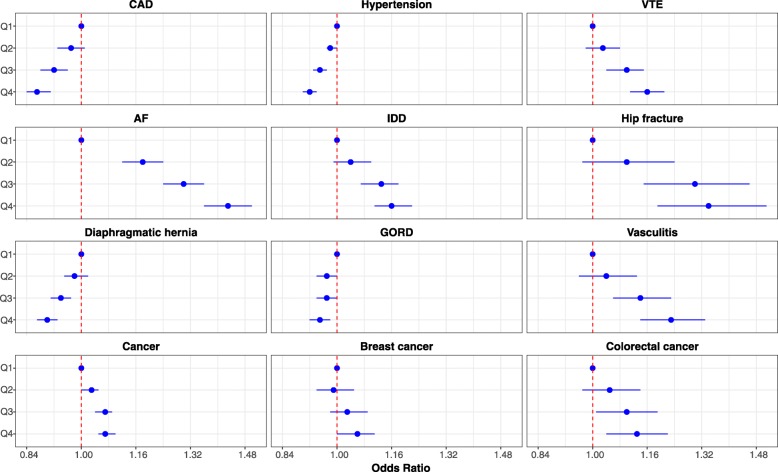
Risk of disease by quartiles of weighted genetic score for height. Legend: CAD coronary artery disease, *VTE* venous thromboembolism, *AF* atrial fibrillation, *IDD* intervertebral disc disorder and *GORD* gastro-oesophageal reflux disease. Associations by quartile of weighted genetic score for height are shown for the 12 diseases which showed an association with genetically determined height (Bonferroni *p* value < 0.05). Individuals in quartile 1 (Q1) (reference quartile) carry the least number, and Q4 carry the highest number of height-increasing alleles. *p* values for trends (GORD *P*_trend_ = 0.003, colorectal cancer *P*_trend_ = 0.003 and breast cancer *P*_trend_ = 0.010, all other diseases *P*_trend_ < 0.001

For the 12 diseases associated with genetically determined height, all exhibited a trend (*p* < 0.05) in either increasing or decreasing risk with carriage of more height-raising alleles (Fig. 2), and the directions were compatible with the findings from the primary genetic analysis. Compared with subjects in Q1, those in Q4 had a decreased risk of CAD (OR = 0.87, 95% CI 0.84–0.91, *p* < 0.001), hypertension (OR = 0.92, 95% CI 0.90–0.94, *p* < 0.001), GORD (OR = 0.95, 95% CI 0.92–0.98, *p* = 0.002) and diaphragmatic hernia (OR = 0.90, 95% CI 0.87–0.93, *p* < 0.001). In contrast, there were increased risks of AF (OR = 1.43, 95% CI 1.36–1.50, *p* < 0.001), VTE (OR = 1.16, 95% CI 1.11–1.21, *p* < 0.001), IDD (OR = 1.16, 95% CI 1.11–1.21, *p* < 0.001), hip fracture (OR = 1.34, 95% CI 1.19–1.51, *p* < 0.001), vasculitis (OR = 1.23, 95% CI 1.14–1.33, *p* < 0.001) and cancer (OR = 1.07, 95% CI 1.05–1.10, *p* < 0.001) along with two specific sites female breast (OR = 1.06, 95% CI 1.004–1.11, *p* = 0.035) and colorectum (OR = 1.13, 95% CI 1.04–1.22, *p* = 0.004) for subjects in Q4 compared with Q1.

### Biological pathways

We identified 202 Ingenuity pathways which included five or more genes from amongst the 691 height-related variants (Additional file 1: Table S6). Table 4 shows the top five pathways associated with each disease. There was little overlap in the pathways showing the strongest association with individual diseases. Furthermore, no individual pathway explains the majority of the association with any disease.

**Table 4:** Top pathways showing the association of height with diseases

Disease	Pathway	Height-associated genes in the pathway	Number of genes	Odds ratio	*p* value	Rank of height pathway#
Coronary artery diseases (CAD)	Caveolar-mediated endocytosis signalling	FLNB, COPA, COPB1, INSR, ITGB8, HLA-C	6	0.50	< 0.001	189
CAD	Production of nitric oxide and reactive oxygen species in macrophages	FGFR2, PIK3R3, PIK3C2A, RHOD, PIK3R1, NFKBIA, MAP2K4, FRS2, GRB2, CREBBP, FGFR4, PRKCZ, MAP3K3	13	0.65	< 0.001	159
CAD	Dendritic cell maturation	FGFR2, COL10A1, PIK3R3, PIK3C2A, PIK3R1, NFKBIA, CREB5, MAP2K4, FRS2, GRB2, HLA-C, CREBBP, TAB1, COL11A2, FGFR4	15	0.66	< 0.001	106
CAD	NGF signalling	FGFR2, PIK3R3, PIK3C2A, RAF1, PIK3R1, TP53, CREB5, MAP2K4, FRS2, GRB2, CREBBP, FGFR4, PRKCZ, MAP3K3	14	0.67	< 0.001	45
CAD	Germ cell-sertoli cell junction signalling	FGFR2, PIK3R3, PIK3C2A, CTNNB1, RHOD, TGFB2, FER, PIK3R1, MAP2K4, FRS2, GRB2, FGFR4, MAP3K3	13	0.69	< 0.001	135
Hypertension	Hepatic cholestasis	MAP2K4, TGFB2, RXRA, INSR, SLCO1C1, FGFR4, PRKCZ, NFKBIA, ESR1, ADCY9	10	0.58	< 0.001	186
Hypertension	RAR activation	PML, PIK3R3, NCOA1, TGFB2, SMAD6, NSD1, BMP2, PIK3R1, SMAD7, RDH14, MAP2K4, SMAD3, RXRA, CREBBP, IGFBP3, PRKCZ, ADCY9	17	0.75	< 0.001	59
Hypertension	IL-1 signalling	GNAS, MAP2K4, GNA12, TAB1, NFKBIA, ADCY9	6	0.57	< 0.001	200
Hypertension	TGF-β signalling	SMAD7, MAP2K4, SMAD3, RAF1, TGFB2, GRB2, SMAD6, CREBBP, TAB1, BMP2, RUNX2	11	0.75	0.001	64
Hypertension	Dopamine-DARPP32 feedback in cAMP signalling	GNAS, ITPR3, CREB5, PRKG1, PRKG2, ITPR1, KCNJ15, KCNJ12, CREBBP, KCNJ16, PRKCZ, ADCY9	12	0.74	0.001	150
Atrial fibrillation	ERK5 signalling	CREB5, GNA12, CREBBP, PRKCZ, MAP3K3, MEF2C, FOXO3	7	1.83	< 0.001	154
Atrial fibrillation	Wnt/β-catenin signalling	LRP5, SOX8, CTNNB1, WNT5A, SOX5, TGFB2, TP53, AXIN2, TLE3, SFRP4, SOX9, CREBBP, TAB1, WNT4	14	1.68	< 0.001	107
Atrial fibrillation	Androgen signalling	GNAS, NCOA1, POLR2A, SMAD3, GNA12, CREBBP, PRKCZ	7	2.14	0.001	197
Atrial fibrillation	Role of Oct4 in mammalian embryonic stem cell pluripotency	REST, FAM208A, RB1, WWP2, TP53, CCNF	6	1.90	0.004	146
Atrial fibrillation	Growth hormone signalling	FGFR2, SOCS5, PIK3R3, PIK3C2A, SOCS2, PIK3R1, IGF2, IGF1R, FRS2, GHR, GRB2, IGFBP3, FGFR4, PRKCZ	14	1.39	0.006	9
Venous thromboembolism (VTE)	Synaptic long-term depression	IGF1R, GNAS, ITPR3, RAF1, PRKG1, GNA12, PRKG2, ITPR1, PRKCZ	9	1.75	< 0.001	192
VTE	Glioma signalling	FGFR2, MTOR, PIK3R3, PIK3C2A, RBL2, RAF1, PIK3R1, TP53, IGF2, IGF1R, FRS2, GRB2, RBL1, CDK6, FGFR4, RB1, PRKCZ	17	1.59	< 0.001	5
VTE	Role of tissue factor in cancer	FGFR2, MTOR, PIK3R3, PIK3C2A, FRS2, GNA12, GRB2, PIK3R1, FGFR4, TP53	10	1.69	< 0.001	155
VTE	Molecular mechanisms of cancer	FGFR2, LRP5, PIK3C2A, PTCH1, WNT5A, RHOD, ARHGEF12, GNA12, SMAD6, BMP2, PIK3R1, MAX, NFKBIA, TP53, CCND3, SMAD7, FRS2, SMAD3, GRB2, BMP6, CREBBP, RBL1, CDK6, FGFR4, PRKCZ, PIK3R3, IHH, CTNNB1, RAF1, TGFB2, GNAS, MAP2K4, TAB1, WNT4, RB1, ADCY9	36	1.37	< 0.001	1
VTE	Role of NFAT in regulation of the immune response	FGFR2, NFATC4, PIK3R3, PIK3C2A, RAF1, GNA12, PIK3R1, NFATC1, NFKBIA, MEF2C, NFATC3, GNAS, ITPR3, FRS2, GRB2, ITPR1, FGFR4, ZAP70	18	1.51	< 0.001	41
Intervertebral disc disorder (IDD)	Protein kinase A signalling	FLNB, AKAP13, PTCH1, HIST1H1E, NFKBIA, PDE11A, SMAD3, ITPR1, PDE3A, CREBBP, PTPDC1, PRKCZ, NFATC4, PTPN14, IHH, CTNNB1, RAF1, TGFB2, NFATC1, ANAPC10, NFATC3, GNAS, ITPR3, CREB5, PTPRG, CDC16, ADCY9, PDE1A	28	1.31	0.005	42
IDD	Wnt/β-catenin signalling	LRP5, SOX8, CTNNB1, WNT5A, SOX5, TGFB2, TP53, AXIN2, TLE3, SFRP4, SOX9, CREBBP, TAB1, WNT4	14	1.49	0.009	107
IDD	PI3K signalling in B lymphocytes	NFATC3, NFATC4, ITPR3, RAF1, PLEKHA1, ITPR1, PIK3R1, NFATC1, PRKCZ, NFKBIA, FOXO3	11	1.80	0.022	132
IDD	VDR/RXR activation	LRP5, NCOA1, TGFB2, RXRA, IGFBP3, PRKCZ, RUNX2	7	1.52	0.023	174
IDD	Factors promoting cardiogenesis in vertebrates	LRP5, CTNNB1, TGFB2, BMP6, BMP2, PRKCZ, MEF2C	7	1.95	0.027	187
Hip fracture	Actin cytoskeleton signalling	FGFR2, PIK3R3, PIK3C2A, SLC9A1, RAF1, ARHGEF12, GNA12, FGF18, PIK3R1, FN1, FRS2, GRB2, FGFR4, SSH2	14	3.46	< 0.001	166
Hip fracture	SAPK/JNK signalling	FGFR2, PIK3R3, PIK3C2A, GNA12, PIK3R1, NFATC1, TP53, NFATC3, MAP2K4, FRS2, GRB2, TAB1, FGFR4, MAP3K3	14	2.56	< 0.001	20
Hip fracture	NRF2-mediated oxidative stress response	FGFR2, FKBP5, PIK3R3, PIK3C2A, MAP2K4, FRS2, RAF1, GRB2, CREBBP, PIK3R1, FGFR4, PRKCZ	12	3.18	0.002	176
Hip fracture	Glucocorticoid receptor signalling	FGFR2, NFATC4, PIK3R3, NCOA1, PIK3C2A, POLR2A, RAF1, TGFB2, PIK3R1, NFATC1, NFKBIA, NFATC3, FKBP5, MAP2K4, FRS2, SMAD3, GRB2, CREBBP, TAB1, FGFR4, ESR1, FOXO3, PRKAB2	23	2.18	0.002	43
Hip fracture	Signalling by rho family GTPases	FGFR2, PIK3R3, PIK3C2A, SLC9A1, RHOD, RAF1, ARHGEF12, GNA12, PIK3R1, GNAS, MAP2K4, FRS2, GRB2, CDC42EP3, FGFR4, PRKCZ	16	2.31	0.003	141
Diaphragmatic hernia	ERK5 signalling	CREB5, GNA12, CREBBP, PRKCZ, MAP3K3, MEF2C, FOXO3	7	0.78	0.013	154
Diaphragmatic hernia	Protein kinase A signalling	FLNB, AKAP13, PTCH1, HIST1H1E, NFKBIA, PDE11A, SMAD3, ITPR1, PDE3A, CREBBP, PTPDC1, PRKCZ, NFATC4, PTPN14, IHH, CTNNB1, RAF1, TGFB2, NFATC1, ANAPC10, NFATC3, GNAS, ITPR3, CREB5, PTPRG, CDC16, ADCY9, PDE1A	28	0.85	0.015	42
Diaphragmatic hernia	Androgen signalling	GNAS, NCOA1, POLR2A, SMAD3, GNA12, CREBBP, PRKCZ	7	0.77	0.015	197
Diaphragmatic hernia	GPCR-mediated integration of enteroendocrine signalling exemplified by an L cell	GNAS, ITPR3, ITPR1, GALR1, ADCY9	5	0.63	0.018	201
Diaphragmatic hernia	SUMOylation pathway	SENP3, RFC1, PML, CTBP2, MAP2K4, RHOD, SENP6, SP3, CREBBP, NFKBIA, TP53	11	0.77	0.025	77
Gastro-oesophageal reflux disease (GORD)	Virus entry via endocytic pathways	FGFR2, FLNB, PIK3R3, PIK3C2A, FRS2, GRB2, ITGB8, HLA-C, PIK3R1, FGFR4, PRKCZ	11	0.69	0.013	90
GORD	HER-2 signalling in breast cancer	FGFR2, PIK3R3, PIK3C2A, FRS2, GRB2, ITGB8, CDK6, PIK3R1, FGFR4, PRKCZ, TP53	11	0.81	0.015	69
GORD	Role of Oct4 in mammalian embryonic stem cell pluripotency	REST, FAM208A, RB1, WWP2, TP53, CCNF	6	0.71	0.018	146
GORD	mTOR signalling	RPS27L, FGFR2, MTOR, PIK3R3, PIK3C2A, RHOD, INSR, PIK3R1, FRS2, GRB2, EIF3H, MLST8, FGFR4, PRKCZ, PRKAB2	15	0.81	0.020	115
GORD	eNOS signalling	FGFR2, LPAR1, PIK3R3, SLC7A1, PIK3C2A, PRKG1, PIK3R1, GNAS, ITPR3, FRS2, GRB2, ITPR1, CCNA2, FGFR4, PRKCZ, ESR1, ADCY9, PRKAB2	18	0.83	0.024	18
Vasculitis	Glioma signalling	FGFR2, MTOR, PIK3R3, PIK3C2A, RBL2, RAF1, PIK3R1, TP53, IGF2, IGF1R, FRS2, GRB2, RBL1, CDK6, FGFR4, RB1, PRKCZ	17	1.82	< 0.001	5
Vasculitis	Molecular mechanisms of cancer	FGFR2, LRP5, PIK3C2A, PTCH1, WNT5A, RHOD, ARHGEF12, GNA12, SMAD6, BMP2, PIK3R1, MAX, NFKBIA, TP53, CCND3, SMAD7, FRS2, SMAD3, GRB2, BMP6, CREBBP, RBL1, CDK6, FGFR4, PRKCZ, PIK3R3, IHH, CTNNB1, RAF1, TGFB2, GNAS, MAP2K4, TAB1, WNT4, RB1, ADCY9	36	1.52	0.002	1
Vasculitis	Growth hormone signalling	FGFR2, SOCS5, PIK3R3, PIK3C2A, SOCS2, PIK3R1, IGF2, IGF1R, FRS2, GHR, GRB2, IGFBP3, FGFR4, PRKCZ	14	1.71	0.004	9
Vasculitis	Chronic myeloid leukemia signalling	FGFR2, PIK3R3, PIK3C2A, RBL2, RAF1, TGFB2, PIK3R1, TP53, CTBP2, FRS2, SMAD3, GRB2, RBL1, CDK6, FGFR4, RB1	16	1.70	0.006	7
Vasculitis	Synaptic long-term depression	IGF1R, GNAS, ITPR3, RAF1, PRKG1, GNA12, PRKG2, ITPR1, PRKCZ	9	1.66	0.006	192
Cancer overall	Adipogenesis pathway	FGFR2, NFATC4, WNT5A, BMP2, CLOCK, TP53, KLF3, EZH2, ARNTL, CTBP2, SMAD3, SOX9, FGFR4	13	1.34	< 0.001	85
Cancer overall	Molecular mechanisms of cancer	FGFR2, LRP5, PIK3C2A, PTCH1, WNT5A, RHOD, ARHGEF12, GNA12, SMAD6, BMP2, PIK3R1, MAX, NFKBIA, TP53, CCND3, SMAD7, FRS2, SMAD3, GRB2, BMP6, CREBBP, RBL1, CDK6, FGFR4, PRKCZ, PIK3R3, IHH, CTNNB1, RAF1, TGFB2, GNAS, MAP2K4, TAB1, WNT4, RB1, ADCY9	36	1.14	0.002	1
Cancer overall	CTLA4 signalling in cytotoxic T lymphocytes	FGFR2, PIK3R3, PIK3C2A, FRS2, GRB2, HLA-C, PIK3R1, FGFR4, ZAP70	9	1.25	0.006	148
Cancer overall	Systemic lupus erythematosus signalling	FGFR2, NFATC4, MTOR, PIK3R3, PIK3C2A, SNRPE, PIK3R1, NFATC1, NFATC3, FRS2, GRB2, HLA-C, FGFR4	13	1.21	0.006	182
Cancer overall	Sphingosine-1-phosphate signalling	S1PR2, FGFR2, PIK3R3, PIK3C2A, FRS2, RHOD, GNA12, GRB2, PIK3R1, FGFR4, ADCY9	11	1.20	0.008	122
Breast cancer	Hypoxia signalling in the cardiovascular system	CREB5, UBE2Z, CREBBP, NFKBIA, TP53	5	0.36	< 0.001	202
Breast cancer	PCP pathway	WNT5A, MAP2K4, ROR2, WNT4, DAAM1	5	2.06	0.038	198
Colorectal cancer	SAPK/JNK signalling	FGFR2, PIK3R3, PIK3C2A, GNA12, PIK3R1, NFATC1, TP53, NFATC3, MAP2K4, FRS2, GRB2, TAB1, FGFR4, MAP3K3	14	2.32	< 0.001	20
Colorectal cancer	Signalling by rho family GTPases	FGFR2, PIK3R3, PIK3C2A, SLC9A1, RHOD, RAF1, ARHGEF12, GNA12, PIK3R1, GNAS, MAP2K4, FRS2, GRB2, CDC42EP3, FGFR4, PRKCZ	16	2.27	< 0.001	141
Colorectal cancer	Small cell lung cancer signalling	FGFR2, PIK3R3, PIK3C2A, PIK3R1, MAX, NFKBIA, TP53, FRS2, GRB2, RXRA, CDK6, FGFR4, RB1	13	2.45	< 0.001	17
Colorectal cancer	Sphingosine-1-phosphate signalling	S1PR2, FGFR2, PIK3R3, PIK3C2A, FRS2, RHOD, GNA12, GRB2, PIK3R1, FGFR4, ADCY9	11	2.47	< 0.001	122
Colorectal cancer	Xenobiotic metabolism signalling	FGFR2, PIK3R3, NCOA1, SMOX, PIK3C2A, RAF1, PIK3R1, MAP2K4, FRS2, GRB2, RXRA, CREBBP, FGFR4, PRKCZ, MAP3K3	15	2.16	< 0.001	185

# Rank indicates the rank of the height pathway as shown in Additional file 1: Table S6; Odds ratio and *p* value indicate the association of the height and the risks of diseases through the pathways

Abbreviations: *cAMP* cyclic adenosine monophosphate, *CLTA4* cytotoxic T-lymphocyte-associated protein 4, *DARPP32* dopamine- and cAMP-regulated phosphoprotein Mr 32 kDa, *eNOS* endothelial nitric oxide synthesis, *ERK5* extracellular signal regulated kinase 5, *GPCR* G protein-coupled receptor, *GTP* guanosine-5′-triphosphate, *HER-2* human epidermal growth factor 2, *JNK* Jun amino terminal kinase, *IL* interlukin, *mTOR* mammalian target of rapamycin, *NFAT* nuclear factor of activated T cells, *NGF* nerve growth factor, *NRF2* nuclear factor erythroid 2–related factor 2, *Oct4* octamer-binding transcription factor 4, *PCP* planar cell polarity, *PI3K* phosphoinositide-3-kinase, *RAR* retinoic acid receptor, *RXR* retinoid X receptor, *SAPK* stress-activated protein kinase, *TGF-β* transforming growth factor beta, *Wnt* wingless-related integration site, *VDR* vitamin D receptor

## Discussion

Our combined epidemiological and genetic analysis showed that adult height is associated with risk of many diseases affecting multiple body systems. We observed a concordance between the epidemiological and genetic analyses for 11 diseases suggesting a primary association between height and risk of these diseases. For colorectal cancer, the genetic analyses suggested a strong association with height but the epidemiological association was slightly weaker. For some diseases (e.g. HF, COPD), we observed an epidemiological association but a much weaker or null genetic association suggesting that the epidemiological associations, despite adjustment for potential confounders, likely remain subject to residual confounding.

We used a large number of genetic variants as instruments in our analysis. It is plausible that some of these variants have effects on disease development that are not linked/mediated through their effects on height (pleiotropy). However, among diseases that showed an association with genetically determined height, we observed trends between carrying more height-raising alleles and disease risk (Fig. 2), evidencing a dose response relationship that supports the role of height, or shared biological mechanisms related to both height and the development of disease.

Our analysis does not exclude the possibility that height itself induces behaviour or reflects circumstances that impact on disease risk. For example, genetically determined height appeared to be associated with a lower Townsend Deprivation Index suggesting the potential impact of genetically determined traits on socio-economic outcomes, which could subsequently impact on disease (Additional file 2: Table S2). However, excluding height-related variants that also associated with Townsend Deprivation Index did not attenuate the observed associations (Additional file 2: Table S3). In the sensitivity analysis, the weighted penalised median produced a weakened association compared with the primary genetic analysis for breast and colorectal cancer, suggesting the possibility of non-homogeneity of the casual effect across variants. Whilst this may lead to incorrect estimate and affect the interpretation of the results, this may not lead to inappropriate inferences [37]. For most diseases in this study, the MR-Egger and weighted median analysis provided consistent estimates with the standard genetic analysis, together with the intercept tests of MR-Egger, supporting the validity of the selected genetic instruments (Table 3).

Turning to individual sets of diseases, the concordant inverse associations between height and risks of CAD in both epidemiological and genetic analyses, together with the absence of any attenuation of the genetic association from exclusion of lipid-related variants [21], suggests a primary impact of shorter height on the vasculature that predisposes to atherosclerotic disease. Our estimate of OR of 0.86 per 1-SD (9.2 cm) increase in genetically determined height agrees with previous reports [21, 22] which showed an estimated equivalent OR of 0.83–0.86. Shorter people have smaller caliber vessels which could cause symptomatic disease despite similar plaque burden [38, 39]. Height also affects pulsatile arterial haemodynamics with increased augmentation of central systolic pressure in shorter people [40] that could influence disease risk in multiple vascular beds.

Our study found evidence of a possible primary association between taller height and risk for AF. Increased atrial size has been recognised as a risk factor of AF [41]. Given the association between body size and left atrial size [42], it is plausible that the height-AF association may be mediated through atrial size. Previous epidemiological studies have reported a positive association between height and VTE [9–11], and our study supports a genetic role of height. One recent study showed an OR = 1.34 per 10 cm increase in genetically determined height [23], which appeared to be strong than our estimate of OR = 1.15 per 9.2 cm increase in genetically determined height, but this study used the genetic risk score (GRS) as the single instrumental variable as opposed to using the variants that comprise the GRS as the instrumental variables in our study and this may lead to the difference in the effect estimates [35]. Taller height is associated with an increased risk of VTE. It is possible that greater venous surface area in taller people increases the risk of VTE.

Extending previous epidemiological studies [43, 44], we found a positive relationship between height and risk of IDD. Whilst the mechanisms remain to be identified, one possible mechanism may be through facet tropism (asymmetry in left and right facet joint angles of lumbar spine) [45]. In addition, our result agrees with a recent meta-analysis of prospective cohort studies which concluded a positive association between height and risk of hip fracture [46]. It is plausible that the association might be mediated with hip axis length, given that height is positively associated with hip axis length [47], which has been reported as a risk factor of hip fracture [48]. Our study also found evidence for a negative association between height and risks of diaphragmatic hernia and GORD. Given that hiatal hernia is the most common type of diaphragmatic hernia, and that it plays an important role in the pathogenesis of GORD [49], it is not surprising height has the same directional impact to the risk of developing these two diseases. Whilst the mechanisms remain unclear, a prior hypothesis is that shorter people have greater intra-abdominal pressure, which increases the risk of developing hiatal hernia and subsequent reflux symptoms [50]. Our study also found strong evidence for a positive association between adult height and vasculitis. This suggests a possible link between height and some aspects of immune function although this requires further investigation. To our knowledge, this is the first analysis performed to evaluate the association of height with risks of diaphragmatic hernia, GORD and vasculitis.

Consistent with previous epidemiological reports [1, 7, 12–15], we found that taller height was associated with a higher overall risk of cancer. One recent study [27] using the UK Biobank showed an OR of 1.10 (95% CI 1.07–1.13) per 1-SD increase in genetically determined height, which appeared to be slightly stronger than our estimate (OR = 1.06, 95% CI 1.04–1.08 per 1-SD in genetically determined height), but one should note the difference in the study designs. Ong et al. [27] excluded self-reported cancer cases, used 2059 genetic variants as instrumental variables and conducted the analysis using the SNP-height and SNP-cancer effects all derived within the UK Biobank. Overlapping subjects in a two-sample MR is known to induce bias [51], and our estimate is potentially more accurate. Nonetheless, these studies strongly suggested that the positive association of height and cancer is primary (not due to confounding), potentially reflecting multiple shared mechanisms influencing cellular growth (Table 4). There were concordant trends towards higher risk with both observed and genetically determined height for various site-specific cancers (Fig. 1f). The diversity of the types of cancers associated with height and their magnitude of associations suggested that there may be different biological mechanisms by which height affects the risks. We observed concordant epidemiological and genetic evidence for breast cancer. Our genetic estimate of OR = 1.07 per 6.2 cm increase (equivalent to OR = 1.12 per 10 cm increase) is similar to previous reports of OR = 1.19 and 1.22 per 10 cm increase in genetically determined height [16, 24]. Our study also agrees with previous studies showing evidence of genetically determined height with risk of colorectal cancer [26], and little evidence with prostate cancer [16, 25]. It is interesting to note that for lung cancer, the genetic and epidemiological associations were in opposite directions, although both associations were not found statistically significant after adjustment for multiple testing suggesting that the observed associations could be due to chance. It is also possible that the epidemiological finding here for lung cancer remains subject to confounding. Our genetic estimate of OR = 1.15 per 9.2 cm increase is consistent with previous report of OR = 1.10 per 10 cm increase in genetically determined height [16]. Previous epidemiological report in women population suggested possible effect modification by smoking status for smoking-related cancers [14], but our epidemiological analysis did not show evidence of difference in height-lung cancer risks between ever and never smokers (interaction of height with smoking status *p* = 0.723).

Our pathway analysis showed that there were different height-associated pathways influencing risks of individual diseases (Table 4). Several of these pathways have been linked to diseases or disease risk in their respective categories, although in many cases, the relationship between the pathways and the disease risks are not very well understood. Nitric oxide signalling has a known relationship to CAD risk [52], and Wnt signalling has been linked to AF [53]. We also found a link between Wnt signalling and IDD, but its role with the disease remains to be investigated. In addition, we observed a link of glioma signalling with VTE and the role of tissue factor in cancer. Several studies suggested possible link of VTE with malignant glioma [54, 55] and other forms of cancer [56]. In this study, we also noted an association of Sphingosine-1-phosphate (S1P) signalling with both the ‘cancer overall’ and colorectal cancer disease categories. S1P is known to have a role in tumorigenesis and tumor growth [57] and has been linked with multiple cancer types and has an association with intestinal inflammation and tumorigenesis [58]. The role of Rho GTPases in the development of colorectal cancer has been reported [59], and this is consistent with our finding of the link of ‘signalling by Rho family GTPases’ pathway with colorectal cancer.

### Limitations of study

Whilst the scale and breadth of the UK Biobank and the ability to examine and directly compare both epidemiological and genetic associations in the same population are particular strengths of our analysis, some limitations need to be highlighted. Although large, the UK Biobank may not be representative of the UK population. There is a skew towards individuals in higher socio-economic groups [30]. Despite a low response rate, the fact that the associations reported in this paper largely agree with other studies, is reassuring. We included both prevalent and incident cases in the primary epidemiological analysis and assumed the exposure of risk factors recorded at baseline remained constant. Our sensitivity analysis revealed generally little impact of the current design as opposed to a prospective design which includes incident cases only. Our design allowed consistent case definition for both genetic and epidemiological analyses and enabled maximum statistical power for detection of association. Finally, there were a small minority of non-White participants in the UK Biobank. We restricted our analysis to individuals of a White ethnic background, and it remains to be shown whether the height-related associations apply to other ethnic groups.

## Conclusion

Adult height is associated with risks of diseases in multiple body systems. Our study, using both epidemiological and genetic approaches, not only confirmed previously reported height associations for CAD, AF, VTE, IDD, hip fracture and cancer, but also identified potential novel associations for GORD, diaphragmatic hernia and vasculitis. It suggests complex relationship between adult height and risk of diseases and shared biological mechanisms underpinning many of the observed height-disease associations.

## Additional files

Additional file 1: **Table S1.** Case definition and data coverage. **Table S6.** Pathways identified by height-associated variants. (XLSX 45 kb)

Additional file 2: **Table S2.** Association of genetically determined height and disease risk factors. **Table S3.** Association of genetically determined height and risks of diseases excluding SNPs with potential pleiotropic effects. **Table S4.** Sensitivity analysis for impact of self-reported cases. **Table S5.** Sensitivity analysis for impact of including prevalent in the case definition for epidemiological analysis. (DOCX 58 kb)

## Abbreviations

AF: Atrial fibrillation
AS: Aortic valve stenosis
BMI: Body mass index
CAD: Coronary artery disease
CI: Confidence interval
COPD: Chronic obstructive pulmonary disease
GORD: Gastro-oesophageal reflux disease
GRS: Genetic risk score
GWAS: Genome-wide association studies
HES: Hospital Episode Statistics
HF: Heart failure
IBD: Inflammatory bowel disease
IBS: Irritable bowel syndrome
ICD: International Classification of Diseases
IDD: Intervertebral disc disorder
MR: Mendelian Randomization
MS: Multiple sclerosis
PVD: Peripheral vascular disease
OR: Odds ratio
SBP: Systolic blood pressure
SNP: Single nucleotide polymorphisms
SD: Standard deviation
VTE: Venous thromboembolism
WHR: Waist-hip-ratio
