# Supplementary material for: Adult height and risk of 50 diseases: a combined epidemiological and genetic analysis
Source: BMC Med. 2018 Oct 25;16:187. doi: 10.1186/s12916-018-1175-7 (PMC6201543; doi:10.1186/s12916-018-1175-7)
Supplement: Supplementary file 2 — Table S2. Association of genetically determined height and disease risk factors. Table S3. Association of genetically determined height and risks of diseases excluding SNPs with potential pleiotropic effects. Table S4. Sensitivity analysis for impact of self-reported cases. Table S5. Sensitivity analysis for impact of including prevalent in the case definition for epidemiological analysis. (DOCX 58 kb) [file 12916_2018_1175_MOESM2_ESM.docx]

**Additional file 2**

Lai FY et al. Adult height and risk of 50 diseases: a combined epidemiological and genetic analysis

| **Supplementary Tables** |  |
| --- | --- |
|  |  |
| **Table S2.** Association of genetically-determined height and disease risk factors | 2 |
| **Table S3.** Association of genetically-determined height and risks of diseases excluding SNPs with potential pleiotropic effects | 3 |
| **Table S4** - Sensitivity analysis for impact of self-reported cases | 4 |
| **Table S5** - Sensitivity analysis for impact of including prevalent in case definition for epidemiological analysis | 6 |

**Table S2. Association of genetically-determined height and disease risk factors**

| Risk Factor | Odds ratio  (95% confidence interval) | | p-value^ |
| --- | --- | --- | --- |
| Obese (BMI >=30) | 0.89 (0.86 to 0.92) | <0.0001* | |
| Waist-hip-ratio | -0.02 (-0.03 to -0.01) | 0.0065 | |
| Townsend deprivation index^#^ | 0.95 (0.93 to 0.98) | <0.0001* | |
| Systolic blood pressure | -0.07 (-0.09 to -0.05) | <0.0001* | |
| Ever smoker | 1.01 (0.99 to 1.03) | 0.3172 | |
| Vigorous activity^##^ | 1.00 (0.98 to 1.02) | 0.7304 | |
| Ever on contraceptive pill | 1.00 (0.98 to 1.03) | 0.7663 | |
| Ever hormone replacement therapy | 1.01 (0.98 to 1.04) | 0.4695 | |
| Nulliparous | 1.10 (1.07 to 1.13) | <0.0001* | |

For binary variables, association is expressed as odds ratios per 1 standard deviation (SD) increase in genetically-determined height, and its 95% confidence interval. For quantitative traits (waist-hip-ratio and systolic blood pressure), the association is expressed as the number of SD change per 1 SD increase in genetically-determined height.

BMI = Body mass index

# Townsend deprivation index - highest quantile

## Vigorous activity - at least once a week for 10+ minutes

**^** p-values shown are unadjusted. * denotes p-value <0.05 after Bonferroni correction for 9 tests.

**Table S3. Association of genetically-determined height and risks of diseases excluding SNPs with potential pleiotropic effects**

|  |  | Excluding those associated with obesity (BMI>30) | Excluding those associated with SBP | Excluding those associated with TPI | Excluding those associated with nulliparity |
| --- | --- | --- | --- | --- | --- |
| Disease | All 691 height SNPs | 489 height SNPs | 429 height SNPs | 619 height SNPs | 624 height SNPs |
| CAD | 0.86 (0.82 - 0.90)*** | 0.92 (0.88 - 0.95)*** | 0.91 (0.86 - 0.97)** | 0.87 (0.83 - 0.91)*** | NA |
| Hypertension | 0.88 (0.85 - 0.91)*** | 0.91 (0.88 - 0.94)*** | 0.97 (0.96 - 0.99)** | 0.88 (0.85 - 0.91)*** | NA |
| AF | 1.33 (1.26 - 1.40)*** | 1.34 (1.25 - 1.42)*** | 1.37 (1.30 - 1.43)*** | 1.33 (1.26 - 1.41)*** | NA |
| VTE | 1.15 (1.11 - 1.19)*** | 1.15 (1.10 - 1.20)*** | 1.17 (1.11 - 1.22)*** | 1.13 (1.09 - 1.17)*** | NA |
| GORD | 0.94 (0.92 - 0.97)*** | 0.96 (0.93 - 0.98)** | 0.96 (0.93 - 0.99)* | 0.95 (0.93 - 0.97)*** | NA |
| Diaphragmatic hernia | 0.91 (0.88 - 0.94)*** | 0.91 (0.89 - 0.94)*** | 0.93 (0.88 - 0.97)*** | 0.92 (0.89 - 0.94)*** | NA |
| IDD | 1.14 (1.09 - 1.20)*** | 1.19 (1.14 - 1.25)*** | 1.13 (1.06 - 1.20)*** | 1.13 (1.07 - 1.20)*** | NA |
| Hip fracture | 1.27 (1.17 - 1.39)*** | 1.34 (1.21 - 1.49)*** | 1.32 (1.18 - 1.48)*** | 1.33 (1.21 - 1.46)*** | NA |
| Vasculitis | 1.20 (1.14 - 1.28)*** | 1.24 (1.16 - 1.33)*** | 1.19 (1.11 - 1.28)*** | 1.16 (1.09 - 1.23)*** | NA |
| Cancer | 1.06 (1.04 - 1.08)*** | 1.05 (1.02 - 1.07)*** | 1.06 (1.04 - 1.09)*** | 1.06 (1.04 - 1.08)*** | NA |
| Colorectal cancer | 1.11 (1.05 - 1.18)*** | 1.09 (1.02 - 1.18)* | 1.12 (1.03 - 1.21)** | 1.11 (1.04 - 1.18)** | NA |
| Breast cancer^ | 1.07 (1.03 - 1.11)*** | 1.05 (1.00 - 1.10)* | 1.06 (1.01 - 1.12)* | 1.06 (1.01 - 1.10)** | 1.05 (1.01 - 1.10)* |

Association is expressed as are odds ratios per 1 standard deviation increase in genetically-determined height, and its 95% confidence interval (***p <0.001, **p<0.01, *p<0.05).

CAD = Coronary Artery Disease; AF = Atrial Fibrillation, VTE = Venous Thromboembolism, GORD = Gastro-oesophageal Reflux Disease, IDD = Intervertebral Disc Disorder. BMI = body mass index; SBP = systolic blood pressure; TPI=Townsend deprivation index; NA = not applicable

**Table S4. Sensitivity analysis for impact of self-reported cases**

|  |  |  | ***Epidemiological analysis*** | | | | | | | | | ***Genetic analysis*** | | | | | | | |
| --- | --- | --- | --- | --- | --- | --- | --- | --- | --- | --- | --- | --- | --- | --- | --- | --- | --- | --- | --- |
|  | no of | registry only | Cases defined by self-report and registry data | | | | | Cases defined by  registry data only | | | | Cases defined by self-report and registry data | | | | Cases defined by  registry data only | | | |
|  | cases | cases | OR* (95% CI) | | p-value^#^ | | | OR* (95% CI) | | p-value^#^ | | OR* (95% CI) | | p-value^#^ | | OR* (95% CI) | p-value^#^ | | |
| **Cardiovascular** |  |  | |  | |  |  | |  | |  | |  | |  | | |  |  |
| Coronary artery diseases (CAD) | 21113 | 17990 | | 0.80 (0.78 - 0.81) | | <0.0001 | 0.79 (0.77 - 0.81) | | <0.0001 | | 0.86 (0.82 - 0.90) | | <0.0001 | | 0.84 (0.80 - 0.88) | | | <0.0001 |  |
| Atrial fibrillation (AF) | 15328 | 14782 | | 1.42 (1.38 - 1.45) | | <0.0001 | 1.41 (1.38 - 1.44) | | <0.0001 | | 1.33 (1.26 - 1.40) | | <0.0001 | | 1.33 (1.26 - 1.40) | | | <0.0001 |  |
| Heart failure (HF) | 5696 | 5601 | | 0.92 (0.89 - 0.96) | | <0.0001 | 0.92 (0.89 - 0.96) | | <0.0001 | | 1.04 (0.98 - 1.10) | | 0.1630 | | 1.04 (0.98 - 1.10) | | | 0.1605 |  |
| Peripheral vascular disease (PVD) | 3926 | 3099 | | 0.77 (0.74 - 0.81) | | <0.0001 | 0.75 (0.71 - 0.79) | | <0.0001 | | 0.93 (0.87 - 1.00) | | 0.0359 | | 0.92 (0.85 - 0.99) | | | 0.0326 |  |
| Venous thromboembolism (VTE) | 15086 | 7590 | | 1.18 (1.16 - 1.21) | | <0.0001 | 1.23 (1.19 - 1.27) | | <0.0001 | | 1.15 (1.11 - 1.19) | | <0.0001 | | 1.21 (1.15 - 1.27) | | | <0.0001 |  |
| Aortic valve stenosis (AS) | 1791 | 1760 | | 0.83 (0.78 - 0.89) | | <0.0001 | 0.83 (0.78 - 0.89) | | <0.0001 | | 1.00 (0.91 - 1.11) | | 0.9332 | | 0.99 (0.90 - 1.10) | | | 0.9033 |  |
| Hypertension | 226504 | 78341 | | 0.83 (0.82 - 0.84) | | <0.0001 | 0.79 (0.78 - 0.80) | | <0.0001 | | 0.88 (0.85 - 0.91) | | <0.0001 | | 0.86 (0.82 - 0.90) | | | <0.0001 |  |
| Stroke | 10676 | 5886 | | 0.83 (0.81 - 0.85) | | <0.0001 | 0.87 (0.83 - 0.90) | | <0.0001 | | 0.96 (0.92 - 1.00) | | 0.0597 | | 0.95 (0.90 - 1.01) | | | 0.1048 |  |
| **Musculoskeletal** |  |  | |  | |  |  | |  | |  | |  | |  | | |  |  |
| Gout | 7593 | 3259 | | 0.98 (0.95 - 1.01) | | 0.1723 | 0.97 (0.92 - 1.01) | | 0.1658 | | 0.97 (0.92 - 1.02) | | 0.2254 | | 1.03 (0.95 - 1.11) | | | 0.4440 |  |
| Osteoarthritis | 64491 | 44395 | | 0.95 (0.94 - 0.96) | | <0.0001 | 0.97 (0.96 - 0.99) | | 0.0002 | | 1.00 (0.97 - 1.03) | | 0.9756 | | 1.01 (0.97 - 1.05) | | | 0.5147 |  |
| Osteoporosis | 11281 | 6803 | | 0.76 (0.73 - 0.78) | | <0.0001 | 0.75 (0.72 - 0.78) | | <0.0001 | | 0.96 (0.92 - 1.00) | | 0.0564 | | 0.99 (0.94 - 1.04) | | | 0.7247 |  |
| Sciatica | 6035 | 2345 | | 1.00 (0.96 - 1.03) | | 0.8595 | 0.95 (0.89 - 1.00) | | 0.0715 | | 1.06 (1.01 - 1.12) | | 0.0315 | | 1.02 (0.93 - 1.12) | | | 0.6484 |  |
| Intervertebral disc disorder (IDD) | 14835 | 8639 | | 1.15 (1.13 - 1.18) | | <0.0001 | 1.06 (1.03 - 1.09) | | 0.0003 | | 1.14 (1.09 - 1.20) | | <0.0001 | | 1.15 (1.10 - 1.20) | | | <0.0001 |  |
| Hip fracture | 2451 | 2298 | | 1.19 (1.12 - 1.26) | | <0.0001 | 1.20 (1.13 - 1.28) | | <0.0001 | | 1.27 (1.17 - 1.39) | | <0.0001 | | 1.29 (1.18 - 1.41) | | | <0.0001 |  |
| **Psychiatric / Neurological** |  |  | |  | |  |  | |  | |  | |  | |  | | |  |  |
| Anxiety | 11033 | 6014 | | 0.93 (0.90 - 0.95) | | <0.0001 | 0.89 (0.85 - 0.92) | | <0.0001 | | 1.01 (0.97 - 1.05) | | 0.6847 | | 0.99 (0.93 - 1.04) | | | 0.6404 |  |
| Depression | 30613 | 12079 | | 0.94 (0.92 - 0.96) | | <0.0001 | 0.90 (0.88 - 0.93) | | <0.0001 | | 0.99 (0.96 - 1.01) | | 0.3166 | | 1.00 (0.96 - 1.04) | | | 0.9085 |  |
| Bipolar | 1665 | 1077 | | 1.00 (0.93 - 1.07) | | 0.9705 | 0.98 (0.90 - 1.07) | | 0.6396 | | 1.06 (0.96 - 1.18) | | 0.2599 | | 1.12 (0.98 - 1.27) | | | 0.1018 |  |
| Multiple sclerosis | 1713 | 1281 | | 1.01 (0.94 - 1.09) | | 0.7049 | 0.96 (0.89 - 1.04) | | 0.3519 | | 1.10 (1.00 - 1.23) | | 0.0591 | | 1.05 (0.93 - 1.18) | | | 0.4702 |  |
| Epilepsy | 5060 | 3796 | | 0.82 (0.79 - 0.85) | | <0.0001 | 0.80 (0.76 - 0.83) | | <0.0001 | | 0.97 (0.91 - 1.03) | | 0.2941 | | 0.98 (0.91 - 1.05) | | | 0.5474 |  |
| Dementia | 1565 | 1520 | | 0.74 (0.69 - 0.79) | | <0.0001 | 0.73 (0.68 - 0.79) | | <0.0001 | | 0.96 (0.86 - 1.07) | | 0.4651 | | 0.96 (0.86 - 1.07) | | | 0.4235 |  |
| Parkinsons' disease | 1341 | 1185 | | 0.96 (0.88 - 1.04) | | 0.2727 | 0.94 (0.86 - 1.02) | | 0.1228 | | 1.10 (0.98 - 1.24) | | 0.1015 | | 1.10 (0.97 - 1.25) | | | 0.1218 |  |
| **Digestive** |  |  | |  | |  |  | |  | |  | |  | |  | | |  |  |
| Gastro-oesophageal reflux disease (GORD) | 36264 | 23224 | | 0.85 (0.84 - 0.86) | | <0.0001 | 0.85 (0.83 - 0.87) | | <0.0001 | | 0.94 (0.92 - 0.97) | | <0.0001 | | 0.95 (0.93 - 0.98) | | | 0.0012 |  |
| Irritable bowel syndrome (IBS) | 13688 | 5605 | | 0.90 (0.88 - 0.92) | | <0.0001 | 0.88 (0.85 - 0.92) | | <0.0001 | | 0.95 (0.91 - 0.98) | | 0.0039 | | 0.91 (0.86 - 0.97) | | | 0.0018 |  |
| Inflammatory bowel disease (IBD) | 5665 | 4653 | | 0.91 (0.87 - 0.94) | | <0.0001 | 0.91 (0.87 - 0.95) | | <0.0001 | | 0.98 (0.93 - 1.04) | | 0.6006 | | 0.99 (0.93 - 1.06) | | | 0.7765 |  |
| Gallstone | 18542 | 14640 | | 0.98 (0.96 - 1.00) | | 0.0649 | 0.97 (0.94 - 0.99) | | 0.0124 | | 1.02 (0.99 - 1.05) | | 0.2912 | | 1.03 (0.99 - 1.06) | | | 0.1663 |  |
| Peptic ulcer | 11423 | 7645 | | 0.80 (0.78 - 0.83) | | <0.0001 | 0.82 (0.79 - 0.85) | | <0.0001 | | 0.95 (0.92 - 0.99) | | 0.0246 | | 0.98 (0.93 - 1.02) | | | 0.3207 |  |
| Liver cirrhosis | 1797 | 1645 | | 0.76 (0.71 - 0.82) | | <0.0001 | 0.76 (0.71 - 0.82) | | <0.0001 | | 0.94 (0.85 - 1.05) | | 0.2694 | | 0.95 (0.85 - 1.06) | | | 0.3414 |  |
| Appendicitis | 7108 | 3737 | | 1.10 (1.06 - 1.14) | | <0.0001 | 1.06 (1.01 - 1.11) | | 0.0239 | | 1.04 (0.99 - 1.09) | | 0.1312 | | 1.05 (0.98 - 1.13) | | | 0.1461 |  |
| Diaphragmatic hernia | 30857 | 27283 | | 0.81 (0.79 - 0.82) | | <0.0001 | 0.80 (0.79 - 0.82) | | <0.0001 | | 0.91 (0.88 - 0.94) | | <0.0001 | | 0.91 (0.88 - 0.95) | | | <0.0001 |  |
| Inguinal hernia | 17217 | 16160 | | 0.97 (0.95 - 0.99) | | 0.0125 | 0.96 (0.94 - 0.98) | | 0.0006 | | 1.04 (0.98 - 1.09) | | 0.1972 | | 1.04 (0.98 - 1.10) | | | 0.1910 |  |
| **Other non-cancer diseases - Endocrine, Immunological, Respiratory, Eye** | | | | | |  |  | |  | |  | |  | |  | | |  |  |
| Diabetes | 26084 | 19895 | | 0.95 (0.93 - 0.97) | | <0.0001 | 0.94 (0.92 - 0.96) | | <0.0001 | | 0.94 (0.90 - 0.99) | | 0.0222 | | 0.95 (0.90 - 1.00) | | | 0.0434 |  |
| Hyperthyroid | 4476 | 1866 | | 1.07 (1.02 - 1.11) | | 0.0051 | 1.08 (1.01 - 1.15) | | 0.0311 | | 1.10 (1.03 - 1.17) | | 0.0039 | | 1.16 (1.05 - 1.28) | | | 0.0029 |  |
| Hypothyroid | 24267 | 14083 | | 1.03 (1.01 - 1.05) | | 0.0037 | 1.04 (1.01 - 1.06) | | 0.0050 | | 1.03 (0.98 - 1.08) | | 0.2016 | | 1.06 (1.00 - 1.11) | | | 0.0441 |  |
| Vasculitis | 5756 | 4840 | | 1.15 (1.11 - 1.19) | | <0.0001 | 1.19 (1.14 - 1.24) | | <0.0001 | | 1.20 (1.14 - 1.28) | | <0.0001 | | 1.25 (1.18 - 1.33) | | | <0.0001 |  |
| Chronic obstructive pulmonary disease (COPD) | 14605 | 9876 | | 0.81 (0.79 - 0.83) | | <0.0001 | 0.76 (0.74 - 0.78) | | <0.0001 | | 1.01 (0.97 - 1.05) | | 0.5808 | | 1.00 (0.96 - 1.05) | | | 0.9892 |  |
| Asthma | 54078 | 26449 | | 0.90 (0.89 - 0.91) | | <0.0001 | 0.84 (0.83 - 0.86) | | <0.0001 | | 0.97 (0.94 - 1.00) | | 0.0467 | | 0.96 (0.92 - 0.99) | | | 0.0265 |  |
| Glaucoma | 6908 | 4496 | | 0.99 (0.96 - 1.03) | | 0.6136 | 0.98 (0.94 - 1.03) | | 0.4062 | | 1.04 (0.97 - 1.11) | | 0.3132 | | 0.99 (0.93 - 1.06) | | | 0.8139 |  |
| Cataract | 22966 | 20362 | | 1.00 (0.98 - 1.02) | | 0.7153 | 1.00 (0.98 - 1.02) | | 0.8809 | | 1.03 (1.00 - 1.06) | | 0.0458 | | 1.03 (1.00 - 1.07) | | | 0.0435 |  |
| **Cancer** |  |  | |  | |  |  | |  | |  | |  | |  | | |  |  |
| Cancer overall | 66818 | 56917 | | 1.09 (1.08 - 1.11) | | <0.0001 | 1.10 (1.08 - 1.11) | | <0.0001 | | 1.06 (1.04 - 1.08) | | <0.0001 | | 1.07 (1.05 - 1.09) | | | <0.0001 |  |
| Lung cancer | 2232 | 2127 | | 0.93 (0.87 - 0.99) | | 0.0206 | 0.93 (0.87 - 0.99) | | 0.0252 | | 1.15 (1.05 - 1.26) | | 0.0022 | | 1.15 (1.05 - 1.26) | | | 0.0038 |  |
| Colorectal cancer | 5052 | 4689 | | 1.07 (1.03 - 1.11) | | 0.0016 | 1.05 (1.01 - 1.10) | | 0.0165 | | 1.11 (1.05 - 1.18) | | 0.0006 | | 1.09 (1.02 - 1.16) | | | 0.0072 |  |
| Female breast cancer | 13396 | 11971 | | 1.08 (1.06 - 1.10) | | <0.0001 | 1.08 (1.06 - 1.10) | | <0.0001 | | 1.07 (1.03 - 1.11) | | 0.0007 | | 1.06 (1.02 - 1.11) | | | 0.0023 |  |
| Prostate cancer | 7462 | 7229 | | 1.03 (1.00 - 1.05) | | 0.0338 | 1.02 (1.00 - 1.05) | | 0.0667 | | 0.99 (0.94 - 1.04) | | 0.6662 | | 0.99 (0.94 - 1.04) | | | 0.7086 |  |
| Melanoma | 4835 | 3249 | | 1.21 (1.16 - 1.26) | | <0.0001 | 1.24 (1.18 - 1.30) | | <0.0001 | | 1.08 (1.02 - 1.15) | | 0.0108 | | 1.12 (1.04 - 1.21) | | | 0.0023 |  |
| Uterus cancer | 1870 | 1494 | | 1.03 (0.98 - 1.08) | | 0.1875 | 1.06 (1.01 - 1.12) | | 0.0214 | | 1.01 (0.92 - 1.12) | | 0.7758 | | 1.05 (0.94 - 1.17) | | | 0.3859 |  |
| Ovary cancer | 1388 | 1132 | | 1.05 (0.99 - 1.11) | | 0.0810 | 1.07 (1.01 - 1.14) | | 0.0318 | | 1.05 (0.94 - 1.18) | | 0.4112 | | 1.10 (0.97 - 1.25) | | | 0.1278 |  |
| Kidney cancer | 1292 | 1190 | | 1.17 (1.08 - 1.27) | | 0.0001 | 1.17 (1.07 - 1.27) | | 0.0003 | | 1.09 (0.97 - 1.23) | | 0.1595 | | 1.10 (0.97 - 1.24) | | | 0.1437 |  |
| Bladder cancer | 1661 | 1133 | | 1.03 (0.96 - 1.10) | | 0.4158 | 1.02 (0.94 - 1.11) | | 0.6722 | | 1.08 (0.97 - 1.20) | | 0.1643 | | 1.09 (0.96 - 1.23) | | | 0.2031 |  |
| Non-Hodgkin lymphoma | 1997 | 1889 | | 1.19 (1.12 - 1.27) | | <0.0001 | 1.20 (1.12 - 1.28) | | <0.0001 | | 1.13 (1.02 - 1.24) | | 0.0152 | | 1.11 (1.01 - 1.23) | | | 0.0377 |  |
| Leukaemia | 1183 | 1039 | | 1.10 (1.01 - 1.20) | | 0.0236 | 1.14 (1.04 - 1.24) | | 0.0038 | | 1.08 (0.96 - 1.23) | | 0.2073 | | 1.15 (1.01 - 1.32) | | | 0.0344 |  |

*Odds ratios and its 95% confidence interval per 1 standard deviation increase in height (epidemiological analysis) or genetically-determined height (genetic analysis)

# p-values are unadjusted

**Table S5. Sensitivity analysis for impact of including prevalent in case definition for epidemiological analysis**

|  | All cases - Incident + prevalent | | | Cases - Incident only | | | |  |
| --- | --- | --- | --- | --- | --- | --- | --- | --- |
| Disease | Number  of cases | OR* (95% CI) | p-value^#^ | Number  of cases | % of all cases | OR* (95% CI) | p-value^#^ |  |
| ***Cardiovascular*** |  |  |  |  |  |  |  |  |
| Coronary artery diseases (CAD) | 21113 | 0.80 (0.78 - 0.81) | <0.0001 | 7193 | 34% | 0.85 (0.82 - 0.88) | <0.0001 |  |
| Atrial fibrillation (AF) | 15328 | 1.42 (1.38 - 1.45) | <0.0001 | 8822 | 58% | 1.34 (1.30 - 1.39) | <0.0001 |  |
| Heart failure (HF) | 5696 | 0.92 (0.89 - 0.96) | <0.0001 | 3721 | 65% | 0.96 (0.91 - 1.00) | 0.0641 |  |
| Peripheral vascular disease (PVD) | 3926 | 0.77 (0.74 - 0.81) | <0.0001 | 1656 | 42% | 0.80 (0.74 - 0.86) | <0.0001 |  |
| Venous thromboembolism (VTE) | 15086 | 1.18 (1.16 - 1.21) | <0.0001 | 3787 | 25% | 1.19 (1.13 - 1.24) | <0.0001 |  |
| Aortic valve stenosis (AS) | 1791 | 0.83 (0.78 - 0.89) | <0.0001 | 1203 | 67% | 0.85 (0.79 - 0.93) | 0.0003 |  |
| Hypertension | 226504 | 0.83 (0.82 - 0.84) | <0.0001 | 5057 | 2% | 0.89 (0.86 - 0.93) | <0.0001 |  |
| Stroke | 10676 | 0.83 (0.81 - 0.85) | <0.0001 | 3337 | 31% | 0.90 (0.85 - 0.95) | <0.0001 |  |
| ***Musculoskeletal*** |  |  |  |  |  |  |  |  |
| Gout | 7593 | 0.98 (0.95 - 1.01) | 0.1723 | 1287 | 17% | 0.96 (0.89 - 1.04) | 0.3479 |  |
| Osteoarthritis | 64491 | 0.95 (0.94 - 0.96) | <0.0001 | 19603 | 30% | 1.00 (0.97 - 1.02) | 0.7042 |  |
| Osteoporosis | 11281 | 0.76 (0.73 - 0.78) | <0.0001 | 3083 | 27% | 0.80 (0.75 - 0.84) | <0.0001 |  |
| Sciatica | 6035 | 1.00 (0.96 - 1.03) | 0.8595 | 1070 | 18% | 0.93 (0.85 - 1.01) | 0.1014 |  |
| Intervertebral disc disorder (IDD) | 14835 | 1.15 (1.13 - 1.18) | <0.0001 | 4078 | 27% | 1.01 (0.97 - 1.06) | 0.6172 |  |
| Hip fracture | 2451 | 1.19 (1.12 - 1.26) | <0.0001 | 1347 | 55% | 1.27 (1.17 - 1.37) | <0.0001 |  |
| ***Digestive*** |  |  |  |  |  |  |  |  |
| Gastro-oesophageal reflux disease (GORD) | 36264 | 0.85 (0.84 - 0.86) | <0.0001 | 10838 | 30% | 0.87 (0.84 - 0.89) | <0.0001 |  |
| Irritable bowel syndrome (IBS) | 13688 | 0.90 (0.88 - 0.92) | <0.0001 | 1987 | 15% | 0.92 (0.86 - 0.99) | 0.0180 |  |
| Inflammatory bowel disease (IBD) | 5665 | 0.91 (0.87 - 0.94) | <0.0001 | 1092 | 19% | 0.95 (0.87 - 1.03) | 0.2141 |  |
| Gallstone | 18542 | 0.98 (0.96 - 1.00) | 0.0649 | 6122 | 33% | 0.97 (0.93 - 1.00) | 0.0888 |  |
| Peptic ulcer | 11423 | 0.80 (0.78 - 0.83) | <0.0001 | 3188 | 28% | 0.87 (0.82 - 0.91) | <0.0001 |  |
| Liver cirrhosis | 1797 | 0.76 (0.71 - 0.82) | <0.0001 | 892 | 50% | 0.82 (0.75 - 0.90) | <0.0001 |  |
| Appendicitis | 7108 | 1.10 (1.06 - 1.14) | <0.0001 | 1186 | 17% | 1.07 (0.99 - 1.17) | 0.0896 |  |
| Diaphragmatic hernia | 30857 | 0.81 (0.79 - 0.82) | <0.0001 | 12588 | 41% | 0.83 (0.81 - 0.85) | <0.0001 |  |
| Inguinal hernia | 17217 | 0.97 (0.95 - 0.99) | 0.0125 | 6475 | 38% | 0.98 (0.94 - 1.01) | 0.1842 |  |
| ***Psychiatric / Neurological*** |  |  |  |  |  |  |  |  |
| Anxiety | 11033 | 0.93 (0.90 - 0.95) | <0.0001 | 4071 | 37% | 0.91 (0.87 - 0.95) | <0.0001 |  |
| Depression | 30613 | 0.94 (0.92 - 0.96) | <0.0001 | 4868 | 16% | 0.91 (0.88 - 0.95) | <0.0001 |  |
| Bipolar | 1665 | 1.00 (0.93 - 1.07) | 0.9705 | 302 | 18% | 0.91 (0.77 - 1.07) | 0.2412 |  |
| Multiple sclerosis | 1713 | 1.01 (0.94 - 1.09) | 0.7049 | 192 | 11% | 0.98 (0.80 - 1.21) | 0.8606 |  |
| Epilepsy | 5060 | 0.82 (0.79 - 0.85) | <0.0001 | 999 | 20% | 0.89 (0.81 - 0.98) | 0.0145 |  |
| Dementia | 1565 | 0.74 (0.69 - 0.79) | <0.0001 | 1276 | 82% | 0.75 (0.69 - 0.81) | <0.0001 |  |
| Parkinsons' disease | 1341 | 0.96 (0.88 - 1.04) | 0.2727 | 564 | 42% | 0.98 (0.87 - 1.11) | 0.7978 |  |
| ***Other non-cancer diseases - Endocrine, Immunological, Respiratory, Eye*** | |  |  |  |  |  |  |  |
| Diabetes | 26084 | 0.95 (0.93 - 0.97) | <0.0001 | 5370 | 21% | 0.96 (0.92 - 0.99) | 0.0262 |  |
| Hyperthyroid | 4476 | 1.07 (1.02 - 1.11) | 0.0051 | 795 | 18% | 1.10 (0.99 - 1.22) | 0.0789 |  |
| Hypothyroid | 24267 | 1.03 (1.01 - 1.05) | 0.0037 | 3144 | 13% | 1.03 (0.98 - 1.09) | 0.2059 |  |
| Vasculitis | 5756 | 1.15 (1.11 - 1.19) | <0.0001 | 2374 | 41% | 1.13 (1.06 - 1.20) | <0.0001 |  |
| Chronic obstructive pulmonary disease (COPD) | 14605 | 0.81 (0.79 - 0.83) | <0.0001 | 5236 | 36% | 0.78 (0.75 - 0.81) | <0.0001 |  |
| Asthma | 54078 | 0.90 (0.89 - 0.91) | <0.0001 | 3652 | 7% | 0.89 (0.85 - 0.94) | <0.0001 |  |
| Glaucoma | 6908 | 0.99 (0.96 - 1.03) | 0.6136 | 2108 | 31% | 0.95 (0.89 - 1.01) | 0.1116 |  |
| Cataract | 22966 | 1.00 (0.98 - 1.02) | 0.7153 | 12283 | 53% | 0.98 (0.95 - 1.01) | 0.1105 |  |
| ***Cancer*** |  |  |  |  |  |  |  |  |
| Cancer overall | 66818 | 1.09 (1.08 - 1.11) | <0.0001 | 24674 | 37% | 1.08 (1.06 - 1.10) | <0.0001 |  |
| Lung cancer | 2232 | 0.93 (0.87 - 0.99) | 0.0206 | 1834 | 82% | 0.94 (0.88 - 1.01) | 0.0744 |  |
| Colorectal cancer | 5052 | 1.07 (1.03 - 1.11) | 0.0016 | 2559 | 51% | 1.05 (0.99 - 1.12) | 0.0741 |  |
| Female breast cancer | 13396 | 1.08 (1.06 - 1.10) | <0.0001 | 4038 | 30% | 1.11 (1.07 - 1.15) | <0.0001 |  |
| Prostate cancer | 7462 | 1.03 (1.00 - 1.05) | 0.0338 | 4333 | 58% | 1.03 (1.00 - 1.06) | 0.0983 |  |
| Melanoma | 4835 | 1.21 (1.16 - 1.26) | <0.0001 | 1242 | 26% | 1.21 (1.12 - 1.32) | <0.0001 |  |
| Uterus cancer | 1870 | 1.03 (0.98 - 1.08) | 0.1875 | 658 | 35% | 1.08 (1.00 - 1.17) | 0.0591 |  |
| Ovary cancer | 1388 | 1.05 (0.99 - 1.11) | 0.0810 | 507 | 37% | 1.10 (1.00 - 1.20) | 0.0402 |  |
| Kidney cancer | 1292 | 1.17 (1.08 - 1.27) | 0.0001 | 702 | 54% | 1.08 (0.97 - 1.21) | 0.1469 |  |
| Bladder cancer | 1661 | 1.03 (0.96 - 1.10) | 0.4158 | 553 | 33% | 1.03 (0.92 - 1.17) | 0.5747 |  |
| Non-Hodgkin lymphoma | 1997 | 1.19 (1.12 - 1.27) | <0.0001 | 944 | 47% | 1.25 (1.14 - 1.37) | <0.0001 |  |
| Leukaemia | 1183 | 1.10 (1.01 - 1.20) | 0.0236 | 557 | 47% | 1.20 (1.07 - 1.36) | 0.0025 |  |

*Odds ratios and its 95% confidence interval per 1 standard deviation increase in height # p-values are unadjusted
